# Transketolase and vitamin B1 influence on ROS-dependent neutrophil extracellular traps (NETs) formation

**DOI:** 10.1371/journal.pone.0221016

**Published:** 2019-08-15

**Authors:** Donporn Riyapa, Darawan Rinchai, Veerachat Muangsombut, Chayanin Wuttinontananchai, Mohammed Toufiq, Damien Chaussabel, Manabu Ato, Jenefer M. Blackwell, Sunee Korbsrisate

**Affiliations:** 1 Center for Research and Innovation, Faculty of Medical Technology, Mahidol University, Nakhon Pathom, Thailand; 2 Systems Biology and Immunology Department, Sidra Medicine, Doha, Qatar; 3 Immunology, Inflammation & Metabolism Department, Sidra Medicine, Doha, Qatar; 4 Department of Immunology, Faculty of Medicine Siriraj Hospital, Mahidol University, Bangkok, Thailand; 5 Department of Immunology, National Institute of Infectious Diseases, Shinjuku, Tokyo, Japan; 6 Telethon Kids Institute, The University of Western Australia, Nedlands, Australia; 7 Department of Pathology, The University of Cambridge, Cambridge, United Kingdom; Hospital for Sick Children, CANADA

## Abstract

Neutrophil extracellular traps (NETs) are a recently identified, web-like, extracellular structure composed of decondensed nuclear DNA and associated antimicrobial granules. NETs are extruded into the extracellular environment *via* the reactive oxygen species (ROS)-dependent cell death pathway participating in inflammation and autoimmune diseases. Transketolase (TKT) is a thiamine pyrophosphate (vitamin B1)-dependent enzyme that links the pentose phosphate pathway with the glycolytic pathway by feeding excess sugar phosphates into the main carbohydrate metabolic pathways to generate biosynthetic reducing capacity in the form of NADPH as a substrate for ROS generation. In this work, TKT was selected as a lead candidate from 24 NET-associated proteins obtained by literature screening and knowledge gap assessment. Consequently, we determined whether TKT influenced NET formation *in vitro*. We firstly established that the release of ROS-dependent NETs was significantly decreased after purified human PMNs were pretreated with oxythiamine, a TKT inhibitor, and in a concentration dependent manner. As a cofactor for TKT reaction, we evaluated the release of NET formation either in vitamin B1 treatment or in combined use of oxythiamine and vitamin B1, and found that those treatments also exerted a significant suppressive effect on the amount of NET-DNA and ROS production. The regulation of TKT by oxythiamine and/or vitamin B1 may therefore be associated with response to the modulation of NET formation by preventing generation of excessive NETs in inflammatory diseases.

## Introduction

Transketolase (TKT) plays pivotal roles in connecting the pentose phosphate pathway (PPP) to glycolytic intermediates [1, 2]. The PPP is required for ribonucleotide synthesis, and it also serves as the primary source of NADPH production [3]. TKT supports the oxidative phase by converting ribulose 5-phosphate back into glucose 6-phosphate to generate more NADPH, which is a key component in fatty acid synthesis and reactive oxygen species (ROS) generation in the metabolic pathway [4, 5]. The reactions catalyzed by TKT in PPP require thiamine pyrophosphate, a biologically active form of thiamine or vitamin B1, and calcium ion (Ca^2+^) as cofactor [2, 6]. It has been reported that the genetic predisposition of Wernicke-Korsakoff syndrome and Alzheimer’s disease are associated with a deficiency of thiamine and functional impairment of the thiamine-dependent enzyme TKT [7].

Thiamine, or vitamin B1, is an essential nutrient used as a dietary supplement and for the treatment of metabolic disorders [8]. Thiamine hydrochloride has been used for a long period of time clinically without reported adverse effects [9–11]. In a previous study, the hydroperoxide generation in linoleic acid peroxidation was significantly decreased by thiamine hydrochloride [12]. Likewise, lipopolysaccharide (LPS)-induced ROS generation in murine macrophages has been prevented by benfotiamine, a unique derivative of thiamine [13]. This analogue has also been found to inhibit expression of inducible nitric oxide synthase (iNOS) and the transcription factor NF-κB in endotoxin-induced uveitis in rats [14].

Polymorphonuclear neutrophils (PMNs) are typically the first effector cells of innate immunity to be recruited to an inflammatory site for containment and clearance of invading pathogens. This function is carried out through the use of different defense strategies [15, 16], including a recently described antimicrobial mechanism involving neutrophil extracellular traps (NETs). The backbone structure of NETs consists of processed chromatin or DNA decorated by specific cytoplasmic proteins from the neutrophilic granules that are extruded into the extracellular environment when PMNs undergo NETosis [17, 18]. Several studies indicate that NADPH oxidase-derived ROS plays an integral part in the reaction cascades that lead to NET release [19]. Experimental inhibition of the peroxidase/ hydrogen peroxide (H_2_O_2_)/halide system by vitamin B1 enhances PMN motility and prevents the oxidation of its membrane *in vitro* and *in vivo* [20, 21]; however, no reports on the involvement of TKT and vitamin B1 in human PMN function, especially NET formation, are available to date.

Given that the PPP and glucose metabolism are known to contribute to NET formation by generating NADPH *via* glucose-6-phosphate dehydrogenase (G6PD) in order to produce superoxide [22], we here investigate the hypothesis that TKT and vitamin B1 play role in ROS-dependent NET formation of human PMNs *in vitro*.

## Materials and methods

### Screening of candidates for follow on investigation and selection of TKT

TKT was listed among 24 NET-associated proteins in a paper by Urban and colleagues [23]. The literature associated with each of the 24 candidate genes was screened by using NCBI’s National Library of Medicine’s PubMed search engine with a query that included the official gene symbol and name as well as aliases of each molecule. For instance in the case of TKT: *“TKT OR transketolase OR HEL107 OR TK1”*. Overlap with the literature relevant to NETs was assessed by adding the following expression to each query: *AND “Neutrophil Extracellular Traps”*. In May of 2017, 2004 papers about TKT were returned after running the query: *TKT OR transketolase OR HEL107 OR TK1*. Candidates for follow on investigation among the 24 NET-associated proteins were selected based on under-representation among the NET literature (exposing the existence of a knowledge gap) [24]. No overlap was found between the TKT and NET literature: *TKT OR transketolase OR HEL107 OR TK1 AND “Neutrophil extracellular traps”* (S1 Table).

### TKT literature profiling

Keywords relevant to six categories corresponding to cell types, diseases, functions, tissues, molecules and processes were identified in titles of the 2004 articles constituting the TKT literature as of May of 2017 and which were returned by the Pubmed query: *“TKT OR transketolase OR HEL107 OR TK1”*. The frequency of articles in the TKT literature containing those keywords in titles and abstracts was subsequently determined and used as a basis to establish a profile of the TKT literature.

### Transcriptome dataset profiling

Relevant studies were identified among a collection of 172 transcriptome datasets hosted on the Benaroya Research Institute’s gene expression browser (GXB). This compendium is comprised of datasets relevant to human immunology and encompasses a total of 12,886 unique transcriptome profiles. It was used to access TKT transcriptional profiles across the datasets available in this resource.

### PMN isolation

Human PMNs were collected from the fresh peripheral blood of healthy donors, as previously described [25, 26]. Whole blood was incubated with an equal volume of 3.0% dextran T-500 (Pharmacosmos, Denmark) and then separated by a Ficoll-Hypaque density gradient centrifugation. Residual red blood cells were removed by hypotonic lysis with sterile distilled water. The PMN viability was >98%, as determined by trypan blue exclusion, and the purity was >95%, as determined by a differential count following Giemsa staining and flow cytometry. Permission was obtained from the Mahidol University Central Institutional Review Board (MU-CIRB), and all human participants provided written informed consent.

### Bacterial preparation

*Staphylococcus aureus* ATCC 25923 was grown on Luria-Bertani (LB) agar for 24 h at 370°C, and single colonies were transferred into LB broth and incubated for 16 h in a shaking incubator. The bacteria were washed twice in phosphate-buffered saline (PBS), and the bacterial concentration was estimated by measuring the absorbance at 600 nm (A600). The viability of the bacteria was confirmed by plating dilutions of the bacterial suspension.

### Quantitative measurement of human TKT

Purified PMNs at a concentration of 2×10^7^ cells/ml were stimulated with 100 nM phorbol 12-myristate 13-acetate or PMA (Sigma, USA) for 90 min at 37°C in a 5% CO_2_ incubator to induce NET formation. The cultures were immediately solubilized with cell extraction buffer. The total solubilized proteins from the supernatants of a 18,000×g centrifugation for 20 min at 4°C were measured by a Bradford protein assay. Human TKT was quantified in PMN extracts containing 10 μg/ml of protein using an ELISA kit (Abcam, UK) and following the manufacturer’s instructions.

### NET quantification

Oxythiamine, a transketolase inhibitor and thiamine hydrochloride (vitamin B1) were purchased from Sigma, USA. Thiamine thiazolone was purchased from Santa Cruz biotechnology, USA. Purified PMNs at a concentration of 2.5×10^5^ cells were pretreated with oxythiamine,thiamine thiazolone, or vitamin B1 at different doses for 60 min or 30 min at 37°C. Moreover, PMNs were separately co-cultured with the combined treatment with oxythiamine and vitamin B1 at different doses at 37°C. The cultures were then stimulated for 90 min with 100 nM PMA as a positive control of NET formation or *S*. *aureus* at a multiplicity of infection (MOI) of 10. The cultures were incubated with restriction enzymes (*Eco*RI and *Hin*dIII, 20 units/ml each, Thermo Scientific, USA) to digest the NET release for 2 h at 37°C, and the reaction was stopped by adding 5 mM EDTA and incubating at 65°C for 15 min. NET-DNA was quantified in the culture supernatant using the Picogreen dsDNA kit (Invitrogen, USA), in accordance with the manufacturer’s instructions.

### Immunofluorescence staining of NETs

Purified PMNs were incubated with 20 mM oxythiamine or 15 mg/ml vitamin B1 prior to stimulation with PMA or *S*. *aureus* for 90 min at 37°C, followed by fixation with 4% (v/v) paraformaldehyde (PFA). Cells were blocked by 3% (w/v) bovine serum albumin (BSA; Sigma, USA) in PBS for 30 min at RT and were then stained with 4'-6-Diamidino-2-phenylindole (DAPI; Molecular Probes, USA), a double-stranded DNA specific vital dye. Stained cells were washed twice with PBS and analyzed by confocal laser scanning microscopy (FluoView FV1000; Olympus, Japan).

### Neutrophil oxidative burst assay

The intracellular ROS was determined by measuring fluorescence of dihydroethidium (DHE; Sigma, USA) according to a previous description [25]. Essentially, after pretreatment either with oxythiamine, thiamine thiazolone, or vitamin B1 alone, or with the combined oxythiamine and vitamin B1, PMNs were stimulated with PMA or *S*. *aureus* for 30 min at 37°C, and 25 μl of a 2,800 ng/ml DHE solution was then added to the cultures. The preparation was incubated at 37°C for 10 min. Cells were washed twice with PBS and fixed with 4% PFA prior to final analysis by using a FACSCanto II (BD Bioscience, San Diego, USA)

### Myeloperoxidase (MPO) activity assay

Purified PMNs at a concentration of 2.5×10^5^ cells were pretreated with vitamin B1 at different doses for 30 min at 37°C following stimulation with 100 nM PMA for 90 min as a positive control of NET formation. We assayed MPO activity, an enzyme occurring nearly exclusively in PMNs, using a commercial kit (Cell Biolabs, USA), according to the manufacturer’s recommended protocol. Briefly, the cultures were incubated with 1 mM hydrogen peroxide (H_2_O_2_) for 30 min at room temperature (RT). A catalase-containing stop solution and chromogen were added. An absorbance was then measured at 405 nm.

### Statistical analysis

All experimental data were presented as mean ± standard error of the mean (SEM). Statistical significance was determined by One-way ANOVA to assess differences between multiple groups, using GraphPad Prism statistical program (GraphPad, San Diego, CA). A *P* value of <0.05 was considered statistically significant.

## Results

### Selection of TKT for downstream investigation the abundance of TKT increases during infections

Among 24 known NET-associated proteins, TKT was selected for follow on investigation based on: 1) The relative under-representation of TKT in the body of literature on NETs; 2) The observation in transcriptome datasets of changes in TKT transcript abundance during infectious processes, and 3) The literature profile obtained for TKT, which indicates relative under-representation of infectious disease studies.

Briefly, relative under-representation of TKT in the NET literature among a list of 24 known NET-associated proteins was determined *via* indexing of the literature for each one of them and checking overlap against the NET literature. No overlap was observed between the NETs and the TKT literature based on a search of title and abstract words, this despite a large body of articles available for this molecule (see methods for details and S1 Fig). Changes in TKT transcript abundance were subsequently observed across transcriptome datasets relevant to human immunology (see methods for details). Profiles of TKT transcript abundance in blood leukocyte populations showed that the abundance of TKT is markedly higher in PMNs and monocytes compared to other cell populations, such as B lymphocytes, CD4 and CD8 T lymphocytes, and natural killer (NK) cells; http://sepsis.gxbsidra.org/dm3/miniURL/view/M3 (S2 Fig).

**Fig 1:**
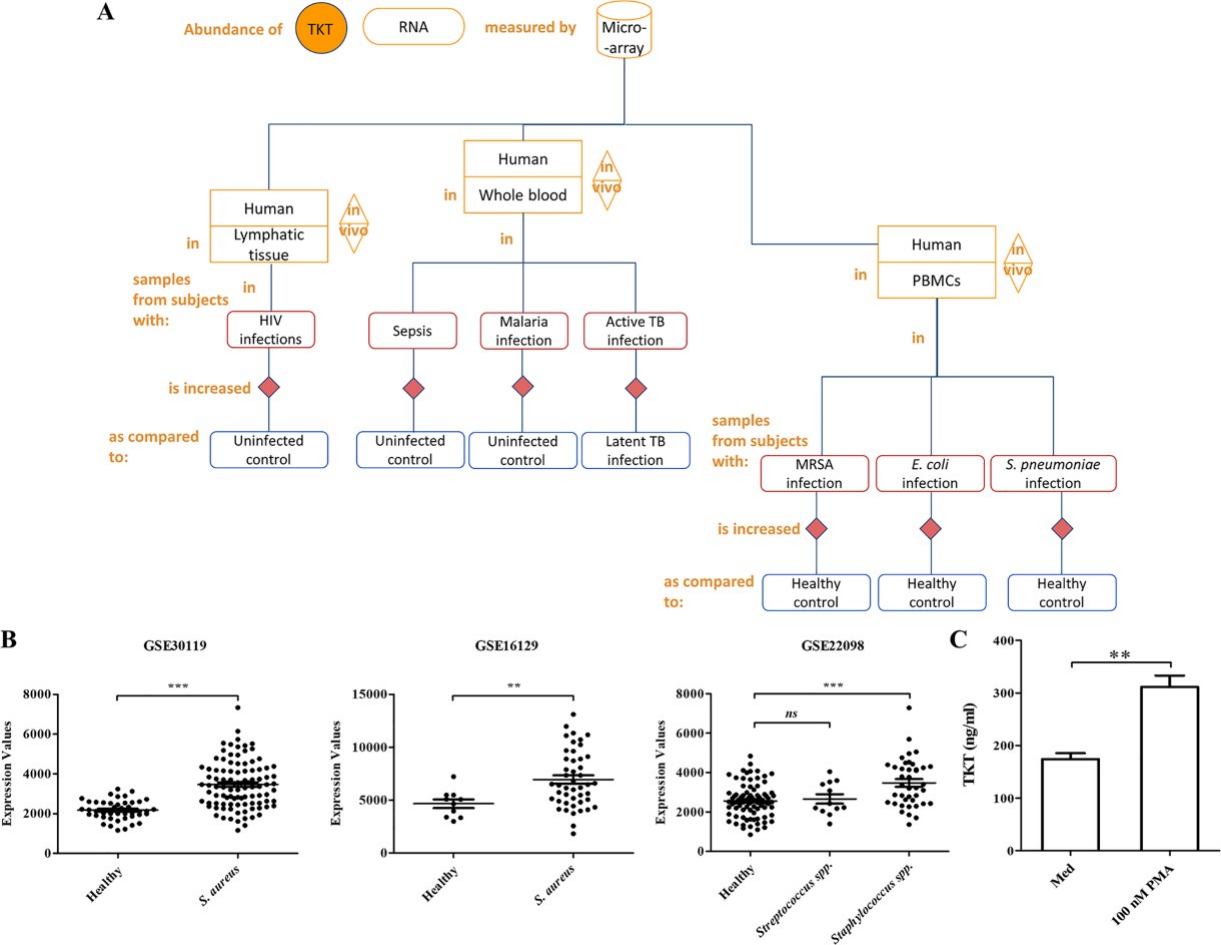
The increased abundance of TKT during infections and NETosis. (A) Flow chart of the abundance of TKT RNA was obtained *via* the screening of a large compendium of transcriptome datasets. The expression of TKT was analyzed in samples from subjects with infections compared to healthy controls or different disease status. (B) Genome-wide analysis of the expressions of TKT transcript response to *S*. *aureus* infection was shown. The information for these plots is provided below: GSE30119: Whole blood samples were collected from patients hospitalized with community-acquired *S*. *aureus* infection and healthy controls. Patients represented the clinical spectrum of acute *S*. *aureus* infections, including skin and soft tissue infections, osteomyelitis, suppurative arthritis, pyomyositis, pneumonia with empyema, and disseminated disease defined as bacteremia [32]. GSE16129: PBMCs from patients with severe *S*. *aureus* infections and healthy controls demonstrated a distinctive and robust TKT expression profile [33]. GSE22098: Whole blood was collected from pediatric patients with *Streptococcus* infections, *Staphylococcus* infections, and healthy controls [30]. (C) The abundance of TKT protein during NETosis was quantified by using ELISA. Purified PMNs were stimulated with 100 nM PMA for 90 min to induce NET formation compared to medium control. Error bar indicates SEM of four independent experiments (n = 4). Statistically significant *P*-values are indicated as follows: **p<0.01, ***p<0.001, and NS-non significant.

Furthermore, the abundance of TKT RNA measured by microarrays in human lymphatic tissue was significantly increased in stage-specific patterns of HIV-1 infection [iFigure/GSE16363] [27]. In whole blood samples, TKT transcript was significantly increased in patients with sepsis [iFigure/GSE13015] [28], malaria infection [iFigure/GSE34404] [29] compared to uninfected controls, and in active pulmonary tuberculosis (TB) compared to latent TB [iFigure/GSE19442] [30]. Transcriptome profiling data also indicated that abundance of TKT RNA was increased in peripheral blood mononuclear cells (PBMCs) during bacterial infections, such as by methicillin-resistant *Staphylococcus aureus* (MRSA), *Escherichia coli*, and *Streptococcus pneumoniae* [iFigure/GSE6269] [31], as illustrated in the flow chart (Fig 1A). Interestingly, three independent transcriptome datasets strongly revealed a significant expression of TKT transcripts in *S*. *aureus* infection compared to healthy controls (Fig 1B), suggesting that TKT might be involved during *S*. *aureus* infection.

### Human TKT protein level increases during NETosis

To address the amount of human TKT protein level observed during NETosis, purified PMNs were stimulated with PMA at a concentration of 100 nM for 90 min to induce NET formation. The results revealed that the human TKT protein level significantly increased during NETosis compared to medium control (Fig 1C).

**Fig 2:**
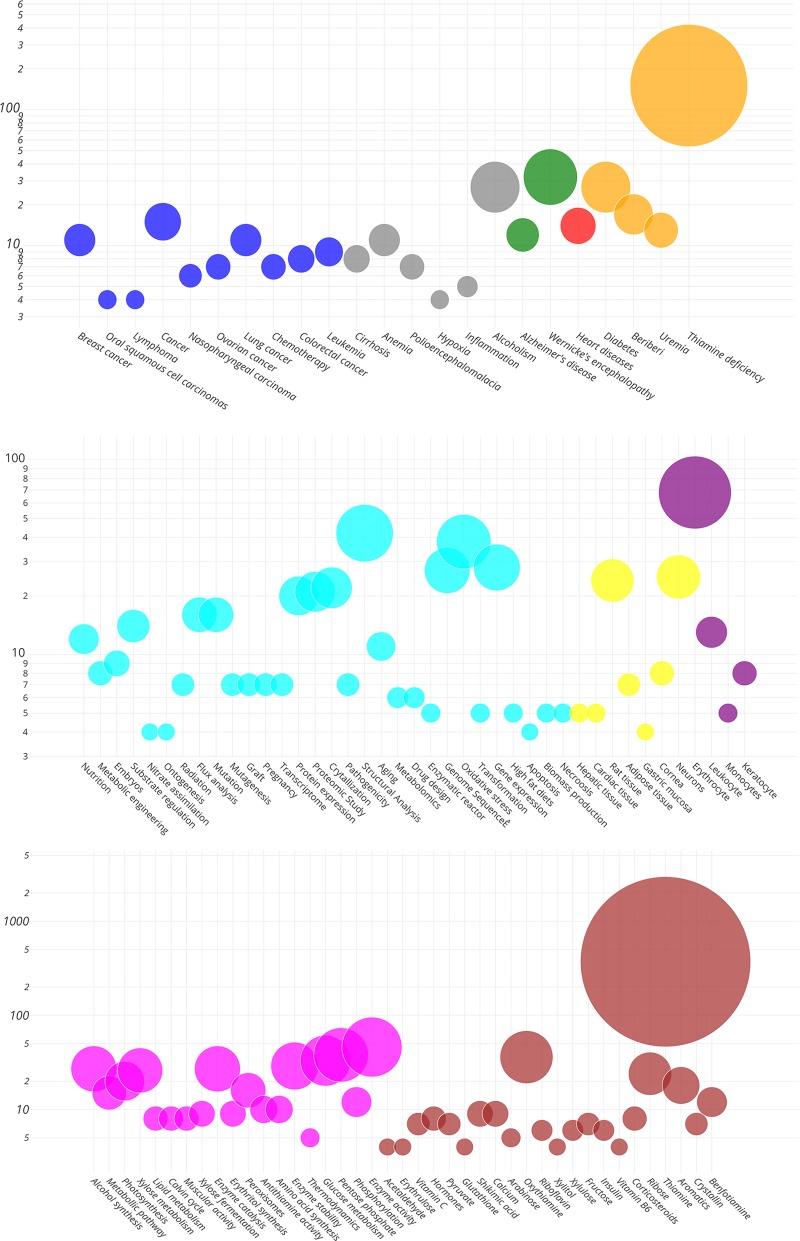
TKT literature profile. By reviewing the literature keywords, the bubble graph showed the frequency of abstracts of papers published on TKT containing curated terms (Y-axis: number of abstract keywords) as of May 2017, and the terms were ordered along the X-axis. The size of the bubbles is proportional to the number of abstract terms.

Finally, profiling of the TKT literature was carried out through manual curation and extraction of keywords in titles of 2004 articles belonging to six different categories: cell types, diseases, functions, tissues, molecules and processes. This provided an overview of the predominant trends in the TKT literature, which is vastly dominated by studies investigating its role in the context of thiamine or vitamin B1 and metabolic disorders linked to vitamin B1 deficiency (Fig 2). Fields of studies in which NETs are known to play an important role, such as autoimmunity and infectious diseases were not represented in the TKT literature.

### NETs are influenced by TKT inhibitor oxythiamine in a dose-dependent manner

**Fig 3:**
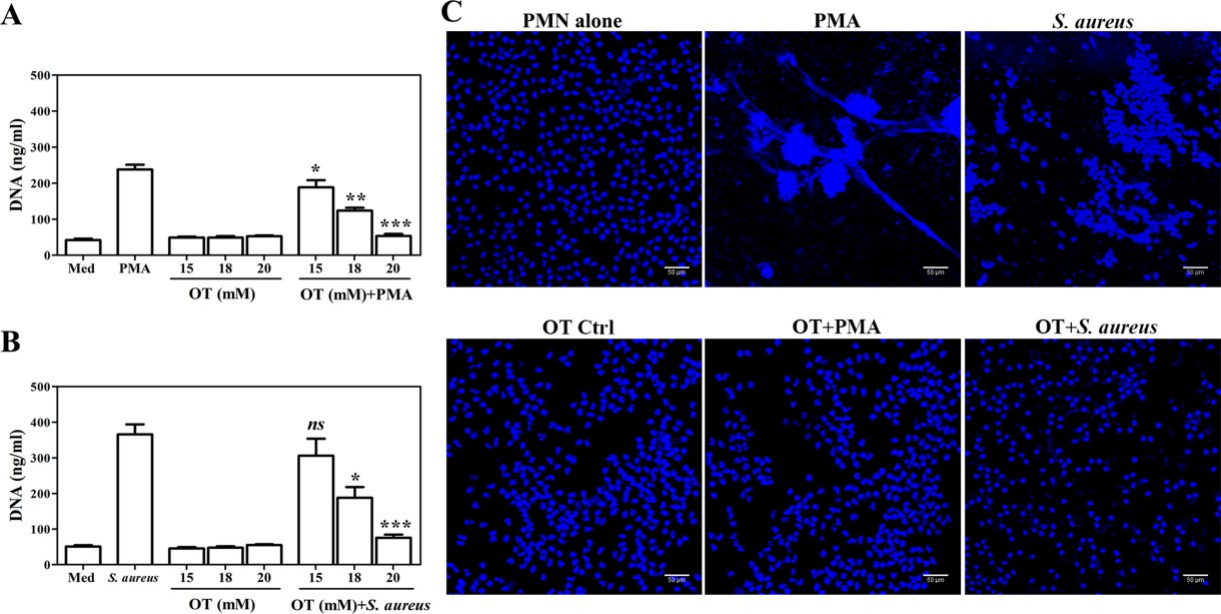
The reduction of NET releases by pretreatment with oxythiamine. PMNs were preincubated with increasing concentrations of oxythiamine, a TKT inhibitor (15, 18, and 20 mM) at 37°C for 60 min. (A) 100 nM PMA or (B) *S*. *aureus* at a MOI of 10 was then added into the cultures at 37°C and 5% CO_2_ for 90 min. The amount of extracellular DNA was quantified using the Picogreen dsDNA kit. The data shown are representative of four independent experiments (n = 4). The horizontal line denotes mean±SEM. Statistically significant *P*-values are indicated as follows: *p<0.05, **p<0.01, ***p<0.001, and NS-non significant. (C) The effect of oxythiamine on NET release by fluorescent images, cells were fixed with 4% PFA and then stained with membrane-permeable DNA-binding dye, DAPI. The representative microscopic images illustrated controls and stimulated PMNs with PMA or *S*. *aureus* after 20 mM oxythiamine treatment. Photomicrographs are of representative cells from three independent experiments. Magnification: 10× or 20×; scale bars: 50 μM. OT; oxythiamine.

To address the role of TKT in NET formation, PMNs were pretreated with different concentrations of oxythiamine as a TKT inhibitor, followed by stimulation with PMA as a positive control of NET induction. Extracellular DNA in culture supernatants was quantified as an indicator of NET formation, as described in the materials and methods section. Doses of this inhibitor were not toxic to the cells, as determined by trypan blue exclusion, and those did not induce the formation of NETs at levels that differed from the medium control (Fig 3). The PMA-induced formation of NETs was reduced by oxythiamine in a dose-dependent and statistically significant manner (Fig 3A).

*S*. *aureus* was selected as a model to confirm whether *S*. *aureus*-induced NETs were influenced by TKT inhibition. This choice was made in part on the basis of the elevated abundance of TKT RNA observed in leukocytes of *S*. *aureus* infected subjects in public transcriptome profiling datasets (Fig 1B). Furthermore, *S*. *aureus* is a bacterium known to uniquely induce NET formation. PMNs release NETs in response to *S*. *aureus*, but this is not due to lysing or breaching the plasma membrane [34]. After oxythiamine pretreatment, the PMNs were infected with *S*. *aureus* at a MOI of 10 for 90 min. The amount of extracellular DNA released by *S*. *aureus*-infected PMNs was significantly decreased in the presence of oxythiamine in a dose-dependent manner, as compared to the *S*. *aureus* control (Fig 3B). These suggest that TKT function may be involved in the formation of NETs.

To confirm whether NET formation was inhibited by oxythiamine after stimulation with PMA or *S*. *aureus*, the confocal micrographs of released extracellular DNA were visualized by DAPI staining. Analysis of pretreatment of PMNs with oxythiamine resulted in a reduction of the released webs of extracellular DNA after stimulation with PMA or *S*. *aureus* compared to the stimulus control by immunofluorescence staining (Fig 3C).

### Vitamin B1 reduces NET formation in a dose-dependent manner

**Fig 4:**
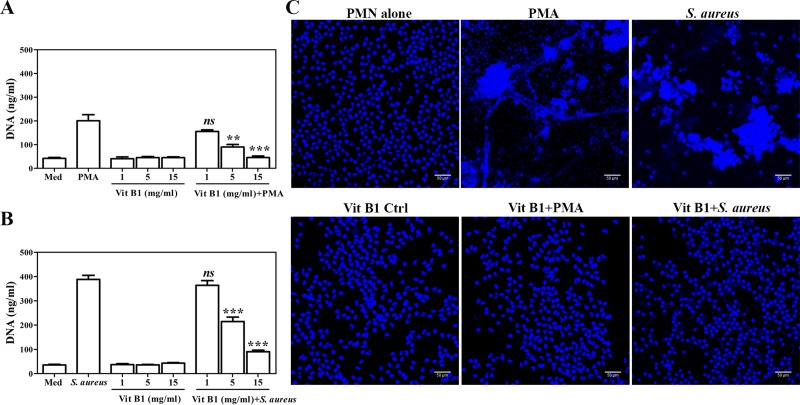
The reduction of NET releases by pretreatment with vitamin B1. Different concentrations of vitamin B1 were added into isolated PMNs at 37°C for 30 min. (A) 100 nM PMA or (B) *S*. *aureus* at a MOI of 10 were then added in the cultures at 37°C and 5% CO_2_ for 90 min. The DNA supernatants were quantified as described above. The data shown are representative of four independent experiments (n = 4). The horizontal line denotes mean±SEM. **p<0.01, ***p<0.001, and NS-non significant. (C) The effect of vitamin B1 on the release of NET formation by fluorescent images, the cultures were fixed with 4% PFA and then stained with DAPI. The confocal images illustrated controls and stimulated PMNs with PMA or *S*. *aureus* after 15 mg/ml vitamin B1 pretreatment. Photomicrographs are of representative cells from three independent experiments. Magnification: 10× or 20×; scale bars: 50 μM.

Thiamine or vitamin B1 was some of the most abundant keywords identified via profiling of the TKT literature, as previously shown (Fig 2). Vitamin B1 has been described as an important cofactor for the TKT reaction, and it acts as an antioxidant in lipid peroxidation [12]. We then determined whether vitamin B1 was also involved in NET formation; PMNs were incubated with vitamin B1 in the doses indicated prior to stimulation with PMA or *S*. *aureus* for 90 min. To examine the toxicity of vitamin B1, purified PMNs were incubated with different concentrations of vitamin B1 for 2 h. Trypan blue exclusion indicated that none of the vitamin B1 at any of the tested doses exerted a toxic effect on PMNs compared with PMN alone, and there was no background of released extracellular DNA in the vitamin B1-pretreated PMNs (Fig 4). The results showed that the formation of NETs was significantly reduced in a manner associated with vitamin B1 at a concentration of 5 mg/ml and 15 mg/ml in both PMA and *S*. *aureus*-stimulated PMNs, as shown in Fig 4A and 4B, respectively.

To confirm the reduction of released webs of extracellular DNA after vitamin B1 pretreatment, the cultured PMNs were stained with DAPI and analyzed by confocal laser scanning microscopy. Immunofluorescence micrographs demonstrated that vitamin B1 could inhibit NET formation in PMA and *S*. *aureus*-stimulated PMNs (Fig 4C), indicating that vitamin B1 treatment also affected the process of NET formation.

### TKT inhibitors abolish level of ROS generation of stimulated PMNs

**Fig 5:**
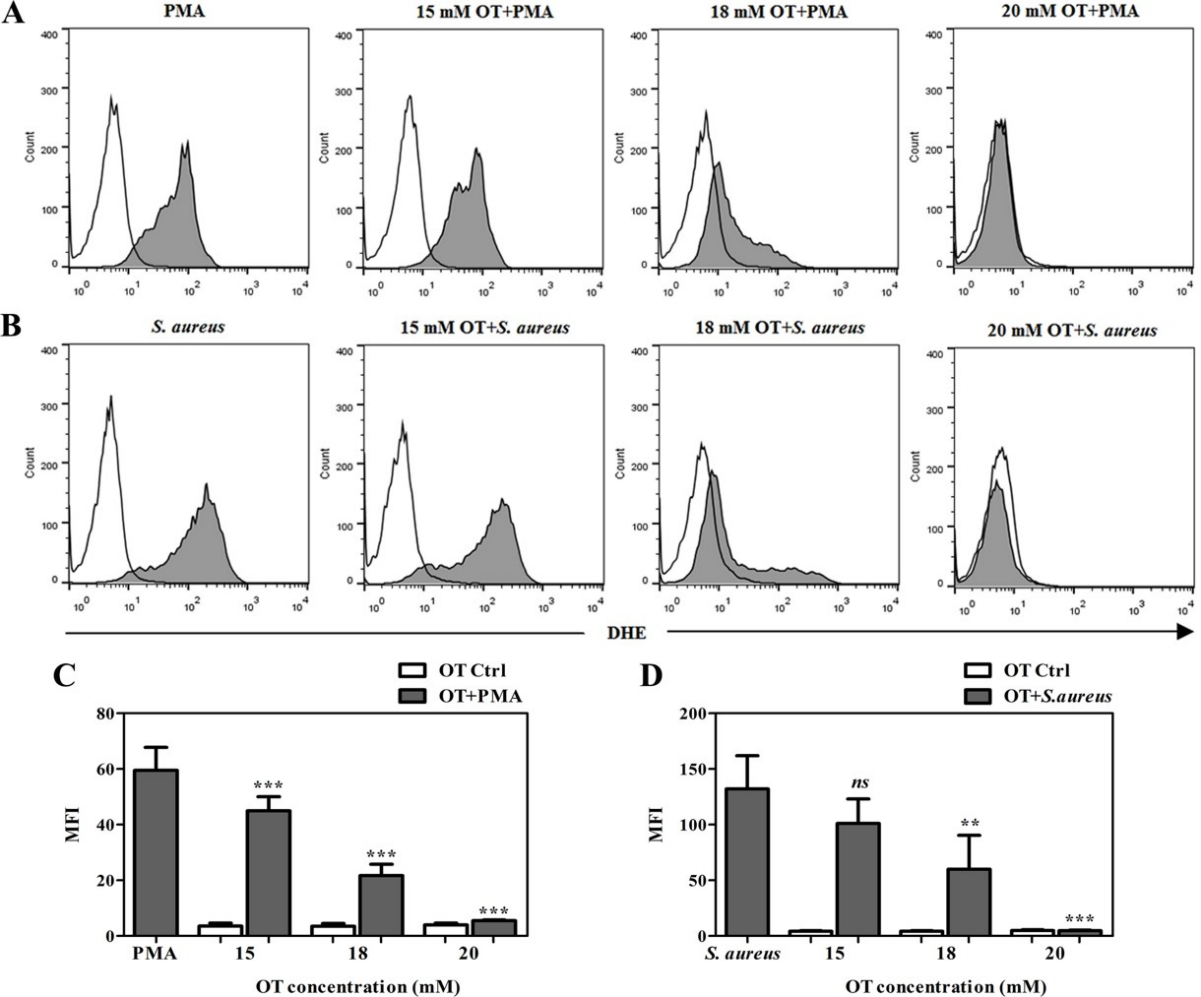
Effect of TKT inhibitor oxythiamine on intracellular ROS production. PMNs were preincubated with or without various concentrations of oxythiamine at 37°C for 60 min. (A, B) Representative images of flow cytometry analysis showed ROS release after PMNs were stimulated with 100 nM PMA or *S*. *aureus* at a MOI of 10, respectively, in the presence or absence of oxythiamine. (C, D) After stimulation with PMA or *S*. *aureus*, the mean fluorescence intensity (MFI) of ROS generation in oxythiamine-treated PMNs compared with positive control was analyzed. Error bar indicates SEM for n = 4–6. **p<0.01, ***p<0.001, and NS-non significant. OT; oxythiamine.

The activity of NADPH oxidase and the development of ROS have been reported as being essential in the formation of NETs [19]. To prove whether oxythiamine is capable of inhibiting NET formation *via* ROS generation, human PMNs were pretreated with oxythiamine at concentrations of 15, 18, and 20 mM for 60 min. The cultures were then stimulated with PMA or *S*. *aureus*, followed by the flow cytometric detection of ROS levels. As seen in Fig 5A and 5C, a significant decrease in the ROS production in PMA-stimulated PMNs was observed for oxythiamine loadings at all concentrations. Meanwhile, the intracellular ROS released by *S*. *aureus*-infected PMNs was significantly reduced by oxythiamine at concentrations of 18 and 20 mM (Fig 5B and 5D), which was consistent with the observation obtained previously with the reduction of the amount of NET-DNA.

**Fig 6:**
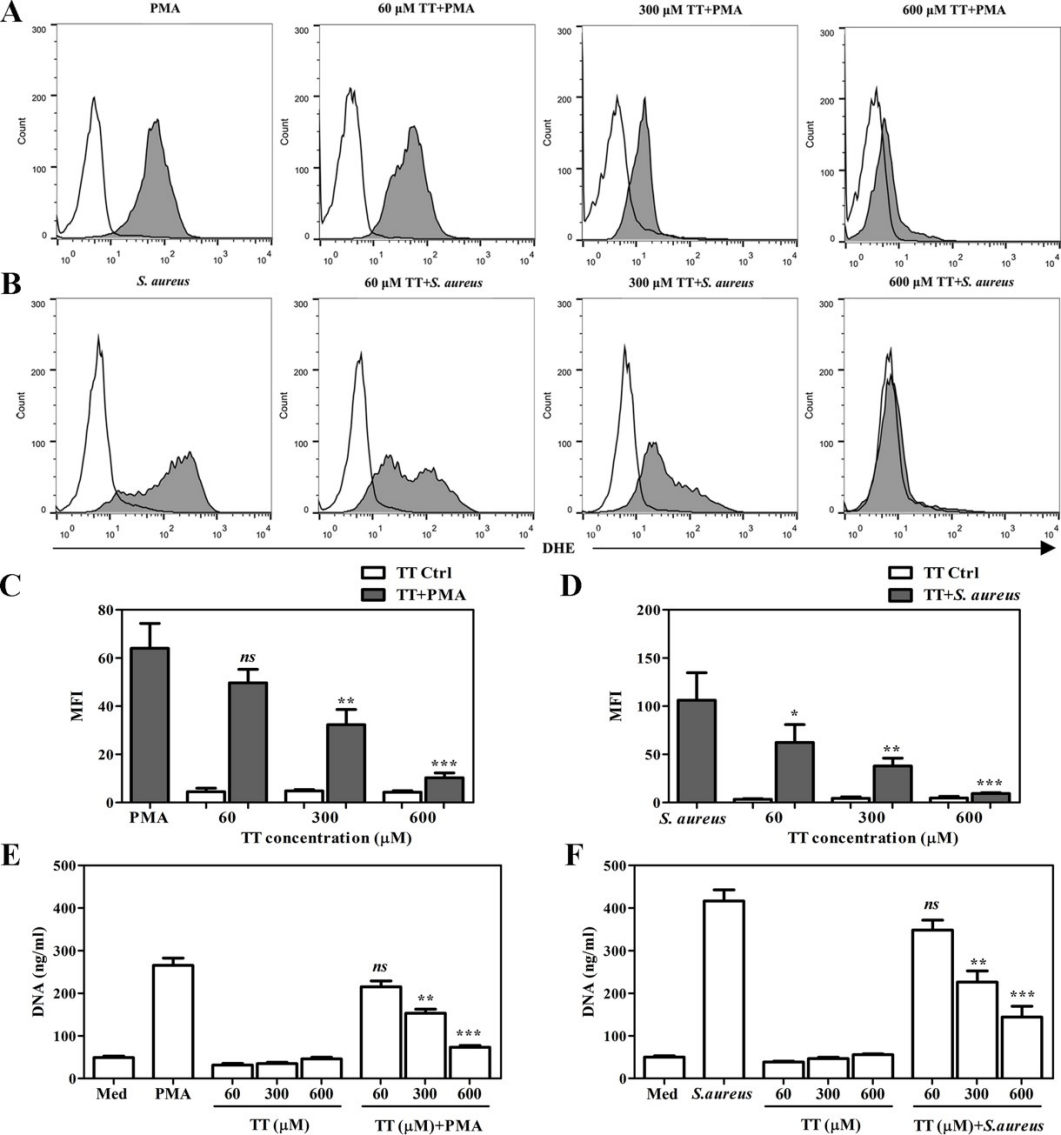
The reductions of ROS levels and NETs after thiamine thiazolone pretreatment. Different concentrations of thiamine thiazolone (60, 300, and 600 μM) were added into isolated PMNs at 37°C for 60 min following stimulation with 100 nM PMA or *S*. *aureus* at a MOI of 10. (A, B) Representative images of flow cytometric detection showed the generation of intracellular ROS in the presence or absence of thiamine thiazolone. (C, D) The mean fluorescence intensity (MFI) of ROS generation in thiamine thiazolone-treated PMNs collected from four healthy individuals was analyzed. (E, F) The amount of NET released from thiamine thiazolone-treated PMNs was quantified. Error bar indicates SEM for n = 4. *p<0.05, **p<0.01, ***p<0.001, and NS-non significant.

We further confirmed the inhibition of TKT function by using thiamine thiazolone to observe reductions of intracellular ROS and NET release. Previously, thiamine thiazolone has been shown to have a very high affinity for the apoenzyme of pyruvate dehydrogenase and other thiamine-utilizing enzymes [35, 36]. We first tested chemical toxicity at all concentrations (60, 300, and 600 μM) by cell viability, using the trypan blue exclusion method, and found no effects on PMN cultures. After pretreatment, the results showed that intracellular ROS production was significantly suppressed by thiamine thiazolone at a stratified dose compared to PMA or *S*. *aureus* stimulation alone (Fig 6A–6D). We also examined the release of extracellular DNA in thiamine thiazolone-pretreated PMNs. The data indicated that NET-DNA was significantly reduced in a manner associated with thiamine thiazolone pretreatment at concentrations of 300 μM and 600 μM in both PMA and *S*. *aureus*-stimulated PMNs (Fig 6E and 6F), confirming that this compound displayed a potent inhibitory activity against TKT activity *in vitro*.

### Vitamin B1 treatment inhibits intracellular ROS generation

**Fig 7:**
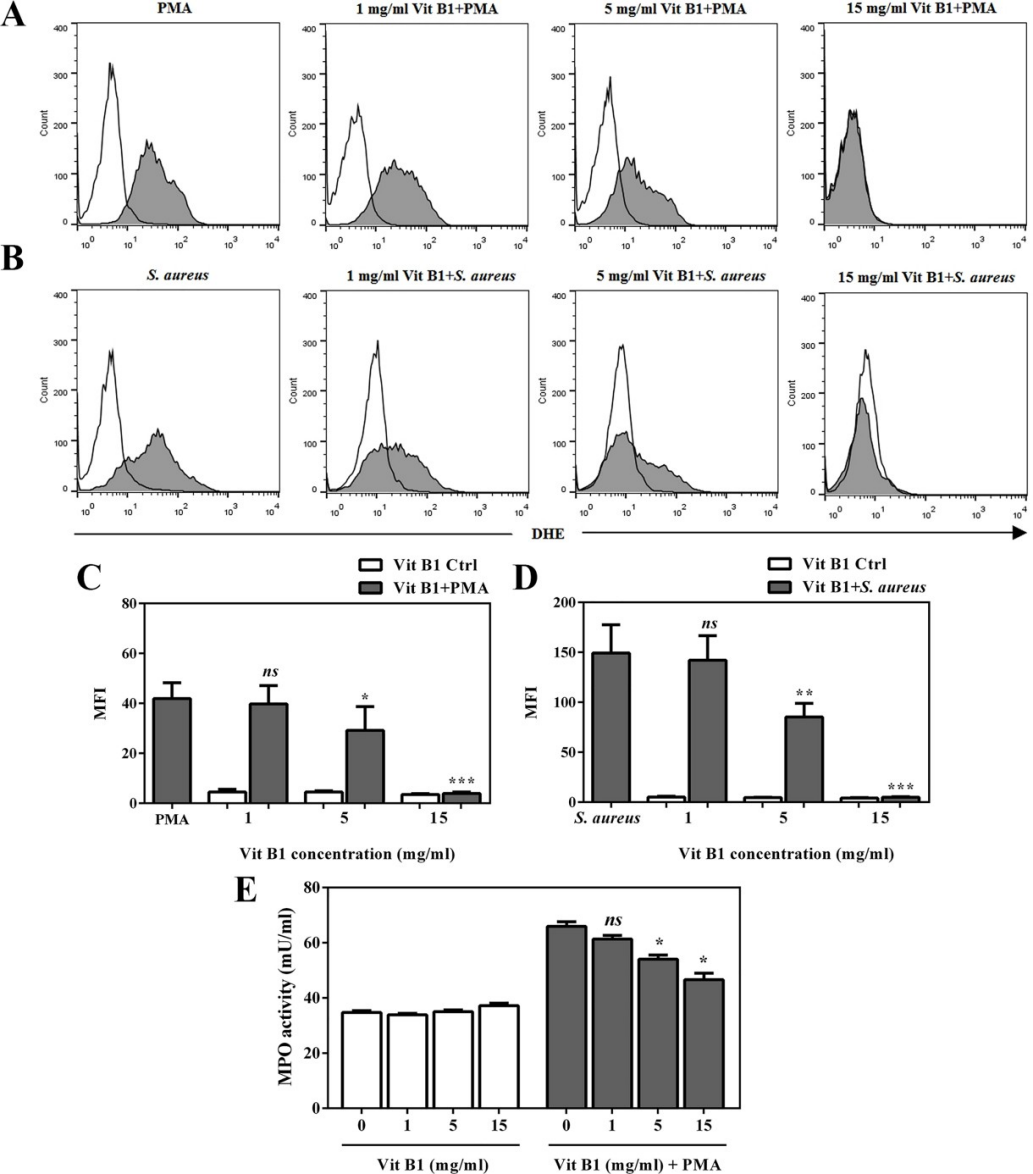
Effect of vitamin B1 on intracellular ROS production. Different concentrations of vitamin B1 were added into purified PMNs at 37°C for 30 min. (A, B) Representative images of flow cytometry analysis showed ROS release after PMNs were stimulated by 100 nM PMA or *S*. *aureus* at a MOI of 10, respectively, in the presence or absence of vitamin B1. (C, D) After stimulation with PMA or *S*. *aureus*, quantitative results showed the MFI of ROS generation in vitamin B1-treated PMNs compared with positive control. (E) The MPO activity was measured in vitamin B1-treated PMNs at different doses for 30 min at 37°C following stimulation with 100 nM PMA for 90 min. The MPO activity was quantified as milliunit/ml (mU/ml). Error bar indicates SEM for n = 3–5. *p<0.05, **p<0.01, ***p<0.001, and NS-non significant.

Furthermore, PMNs were incubated with vitamin B1 at concentrations of 1, 5, and 15 mg/ml for 30 min prior to stimulation with PMA or *S*. *aureus* to address the mechanism of vitamin B1 on ROS production. The results indicated that the level of ROS generation was significantly reduced relative to PMA-stimulated PMNs as a positive control at 5 and 15 mg/ml of vitamin B1 (Fig 7A and 7C). Moreover, the inhibitory effect of vitamin B1 at the same concentrations was observed in *S*. *aureus*-infection PMNs (Fig 7B and 7D), indicating that decreased NETs could be affected by vitamin B1 treatment *via* ROS-mediated cellular signaling.

As previously described, vitamin B1 serves as antioxidant through inhibition of peroxidase/hydrogenperoxide (H_2_O_2_)/halide system in the PMN oxidation process [20, 21]. To clarify whether the dose effects of vitamin B1 caused by its antioxidant effects, vitamin B1-treated PMNs were measured for activity of MPO, a specific granular enzyme of PMNs, which is considered as a marker of stimulated PMNs in oxidative stress by generating oxidant species, particularly hypochlorous acid (HOCl). The results showed that vitamin B1 at concentrations of 5 and 15 mg/ml significantly decreased PMN MPO activity in a dose-dependent manner (Fig 7E), suggesting that the effect of vitamin B1 treatment on ROS production could be regulated *via* its antioxidant property during NETosis.

### Combined treatment with oxythiamine and vitamin B1 inhibits ROS-dependent NETs

**Fig 8:**
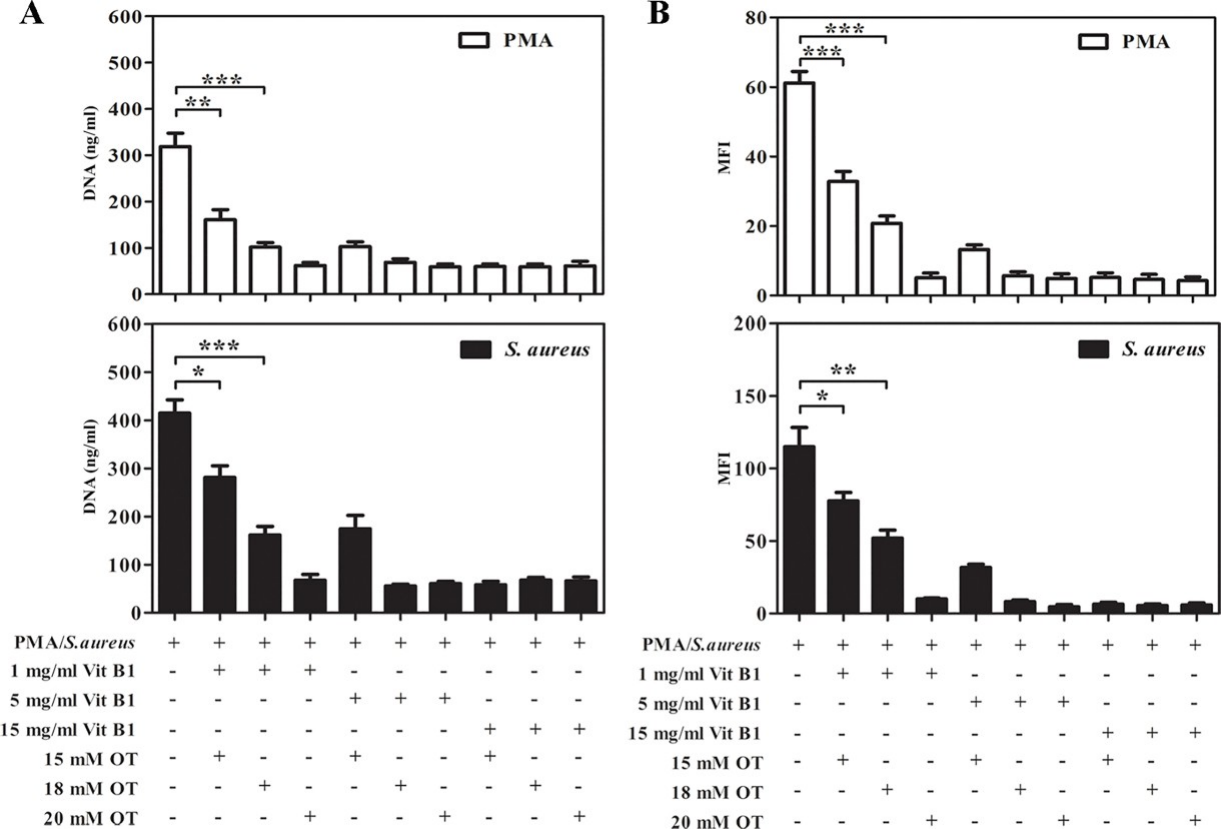
Effect of treatment with TKT inhibitor co-applied with vitamin B1 on NETs and intracellular ROS production. The different doses of oxythiamine and vitamin B1 were co-applied into purified PMNs following stimulation with 100 nM PMA or *S*. *aureus* at a MOI of 10. (A) The amount of extracellular DNA was quantified after human PMNs were stimulated. (B) The relative levels of intracellular ROS generation were presented as the MFI from four healthy individuals. Error bar indicates SEM of four independent experiments. *p<0.05, **p<0.01, and ***p<0.001.

We elucidated the effect of co-treatment of oxythiamine and vitamin B1 at different concentrations on NETs. The results showed that the combined treatment with oxythiamine and vitamin B1 at the minimum doses (15 mM and 1 mg/ml, respectively) significantly improved the reductions of extracellular DNA release and ROS generation (Fig 8A and 8B). In contrast, as shown in the original manuscript, treatment with oxythiamine alone at a concentration of 15 mM, or vitamin B1 alone at a concentration of 1 mg/ml did not affect *S*. *aureus*- and PMA/*S*. *aureus*-mediated NETosis, respectively.

## Discussion

The reactions of TKT provide a link between the PPP and glycolysis by feeding excess sugar phosphates into the main carbohydrate metabolic cascades. Its presence is essential for the production of NADPH to be maintained under different metabolic conditions [2, 37]. TKT has been classified as an energy metabolic enzyme in the cytosolic skeleton of PMNs, which mainly rely on glycolysis for their cellular energy consumption [38, 39]. Likewise, we demonstrated that the expression of TKT transcripts in PMNs and monocytes were found to be markedly increased compared to lymphocyte and NK cell populations in the transcriptome datasets.

A newly recognized activity of PMNs was described in which the leukocytes generate extracellular traps (ETs) [18]. Upon activation with stimuli or microorganisms, PMNs produce large amounts of superoxide *via* NADPH oxidase and superoxide dismutases (SODs), and then convert the superoxide radical to H_2_O_2_ [4]. The ROS generation has been described as an essential signal for NET releases. NETs are lattices of processed DNA and histones decorated with antimicrobial proteins and granule enzymes [19, 40]. Using a proteomic approach, 24 different NET proteins have been identified. TKT belonged to the NET-associated protein localized in the cytoplasm of PMNs [23]. Interestingly, we found that the human TKT protein level significantly increased during NETosis after PMNs were treated with PMA as a positive control of NET induction. In addition, it was demonstrated in our study that the inhibitory action of oxythiamine by blocking TKT activity significantly reduced the level of ROS and NET-DNA stimulated with PMA in a dose-dependent manner. With reference to the transcriptome datasets, TKT RNA was significantly elevated in whole blood or PBMCs from patients with an *S*. *aureus* infection, leading us to investigate whether *S*. *aureus*-induced NET formation was influenced by TKT inhibition. The results indicated that the blocking of TKT by oxythiamine could also significantly decrease ROS production and consequently resulted in a reduction of the NET-DNA released from *S*. *aureus*-infected PMNs. Taken together, these findings support the suggestion that NET formation might be influenced by the reduction of NADPH through TKT inhibition. Evidence implicates that the PPP is controlled by transketolase-like 1 (TKTL1) activity, and the knockdown of TKTL1 leads to a significant decrease of NADPH and ribose-5-phosphate levels in nasopharyngeal carcinoma cells [41]. Additionally, a metabolic shift toward the PPP is necessary for NET release because G6PD, a regulatory enzyme responsible for initial deviation of glucose into the PPP, fuels NADPH for superoxide productions, which induce NET formation [22]. In general, oxythiamine has been widely established to abolish the activity of TKT and provides the effectiveness in anticancer activity *in vitro* and *in vivo* which is also currently in the clinical and pre-clinical phase [42, 43]. Comparing the effective doses of oxythiamine in different types of cancer cells, we found that treatment with oxythiamine possessed inhibition activity in the millimolar range in the reductions of ROS and NET-DNA released by human PMNs. However, we cannot be certain that this directly results in off-target effects in high concentration of oxythiamine-treated human PMNs. We further examined the inhibition of TKT by using thiamine thiazolone which has been reported a significant binding to TKT and shows an inhibitory effect of thiamine-utilizing enzyme activity [36]. Thiamine thiazolone could inhibit the releases of ROS level and NET formation in a concentration dependent manner which possessed low micromolar cellular potency against TKT in human PMNs, demonstrating that thiamine thiazolone provides a potent alternative inhibitor of TKT function.

TKT is a thiamine pyrophosphate (vitamin B1)-dependent enzyme. A decreased TKT activity is presumed to be due to the decrease of vitamin B1 used for thiamine deficiency diagnosis [2, 44]. As referred to in the bubble graph analysis, existing knowledge pertaining to TKT was retrieved using NCBI’s National Library of Medicine’s PubMed search engine. Thiamine or vitamin B1 has so far showed a top hit association within TKT research studies. Vitamin B1 provides many health benefits in its role as an essential nutrient, including energy production and antioxidant activity [45]. Vitamin B1 directly reacts with free radicals and hydroperoxides, undergoes oxidation, and produces antioxidant effects [46]. Thiamine deficiency in a mouse model showed a high level of 4-hydroxy 2-nonenal (4-HNE), a product of ROS-induced lipid peroxidation in the cell membrane [47]. The actions of vitamin B1 also prevent the PMN motility-inhibiting effects of the horseradish peroxidase (HRP)/H_2_O_2_/halide system and protect the neutrophil sulfhydryl groups, an indicator of the state of reduction of the cell [48]. We established that vitamin B1 treatment at concentrations of 5 and 15 mg/ml in human PMNs *in vitro* showed significantly decreased ROS generation, leading to the inhibitory action in PMA or *S*. *aureus*-induced NET release. In particular, vitamin B1 directly affected the intracellular ROS production in PMA-stimulated PMNs *via* its antioxidant effects. Similarly, there is evidence that antioxidants such as vitamins C and D significantly attenuate NETosis activity in PMNs [49, 50]. Although treatment with vitamin B1 alone at concentration of 1 mg/ml did not affect the ROS-dependent NETosis in PMA and *S*. *aureus* stimulation, the combined minimum doses of oxythiamine and vitamin B1 treatment could significantly achieve the reduction in NET forming, suggesting that co-treatment with oxythiamine and vitamin B1 might contribute to the potential regulation of ROS-dependent NETosis.

## Conclusions

Taking into account the results obtained in our study, TKT inhibitors and vitamin B1 could modulate a significant inhibition of ROS-dependent NETosis activity. Moreover, an addition of vitamin B1 in the presence of the TKT inhibitor oxythiamine results in significant inhibition of ROS-dependent NETosis activity that is not observed when either treatment is used alone at the minimum doses. Whilst this seems counter-intuitive given the role of vitamin B1 as a co-factor for TKT activity, the impairment of TKT activity under oxythiamine or thiamin thiazole exposure may be influenced by a change of dynamic metabolic demands in the non-oxidative arm of the PPP, contributing to the abolished NADPH oxidase-derived ROS. In this case, the pharmacological activity of vitamin B1 as an antioxidant appears to be important in this combined effect. Combined treatments with a TKT inhibitor and vitamin B1 may thus pave the way to an alternative therapeutic regime and recommendation for adverse pathological outcomes involving excessive NET formation such as in severe sepsis or auto-inflammatory diseases. Further work is required to understand in more detail the mode of action of this combined therapy and its protective properties as well as to mitigate against any potential negative side effects during treatment.

## Supporting information

S1 Fig Total number of publications of each 24 NET-associated gene. After running query with or without the “NETs” keyword in PubMed, each candidate gene from 24 NET-associated genes was counted.
(DOCX)

S2 Fig The expression of TKT transcript in human blood leukocyte populations. Whole blood samples were collected from healthy donors and patients. PMNs, monocytes, B cells, CD4 T cells, CD8 T cells and NK cells were isolated prior to profiling *via* RNA sequencing. The graph presented an abundance of TKT RNA in each cell population.
(TIF)

S1 Table Lead candidate ranking. Lead candidates were ranked from the 24 NET-associated genes when knowledge gap was exposed.
(DOCX)
