# Supplementary material for: Transketolase and vitamin B1 influence on ROS-dependent neutrophil extracellular traps (NETs) formation
Source: PLoS One. 2019 Aug 15;14(8):e0221016. doi: 10.1371/journal.pone.0221016 (PMC6695114; doi:10.1371/journal.pone.0221016)
Supplement: S1 Fig — After running query with or without the “NETs” keyword in PubMed, each candidate gene from 24 NET-associated genes was counted. (DOCX) [file pone.0221016.s001.docx]

**S1 Fig. Total number of publications of each 24 NET-associated gene.** After running query with or without the “NETs” keyword in PubMed, each candidate gene from 24 NET-associated genes was counted.
