# Supplementary figures and images for: Transketolase and vitamin B1 influence on ROS-dependent neutrophil extracellular traps (NETs) formation
Source: PLoS One. 2019 Aug 15;14(8):e0221016. doi: 10.1371/journal.pone.0221016 (PMC6695114; doi:10.1371/journal.pone.0221016)

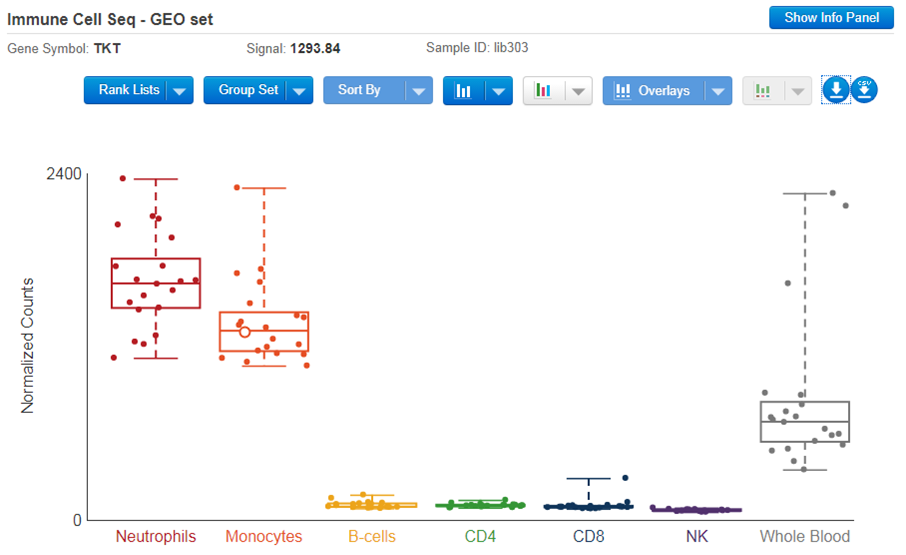

Supplement: S2 Fig — Whole blood samples were collected from healthy donors and patients. PMNs, monocytes, B cells, CD4 T cells, CD8 T cells and NK cells were isolated prior to profiling via RNA sequencing. The graph presented an abundance of TKT RNA in each cell population. (TIF) [file pone.0221016.s002.tif]
