# Supplementary material for: Transketolase and vitamin B1 influence on ROS-dependent neutrophil extracellular traps (NETs) formation
Source: PLoS One. 2019 Aug 15;14(8):e0221016. doi: 10.1371/journal.pone.0221016 (PMC6695114; doi:10.1371/journal.pone.0221016)
Supplement: S1 Table — Lead candidates were ranked from the 24 NET-associated genes when knowledge gap was exposed. (DOCX) [file pone.0221016.s003.docx]

**S1 Table. Lead candidate ranking.** Lead candidates were ranked from the 24 NET-associated genes when knowledge gap was exposed.

**Gene symbol** **Definition Query** **No. of articles No. of articles in**

**in PubMed** **PubMed with NETs**

ELA Elastase ELA OR ELANE OR elastase OR SCN1 OR "PMN-E" [ti] 18341 78

MPO Myeloperoxidase MPO OR myeloperoxidase [ti] 12200 68

H3 Histone H3 H3 OR HIST2H3C OR "histone cluster 2 H3 family member c" 23396 23

OR H3FM OR H3FN [ti]

CTSG Cathepsin G CTSG OR "cathepsin G" OR CATG [ti] 1902 10

PR3 Proteinase 3 PRTN3 OR "proteinase 3" OR PR3 [ti] 2826 8

H4 Histone H4 H4 OR HIST2H4A OR "histone cluster 2 H4 family member a" 11171 5

OR H4FN OR FO108 OR HIST2H4 [ti]

CAT Catalase Catalase [ti] 6506 3

S100A12 S100 calcium binding S100A12 OR "S100 calcium binding protein A12" 615 3

protein A12 OR MRP6 OR ENRAGE [ti]

LYZ Lysozyme LYZ OR lysozyme OR LZM OR LYZF1 [ti] 23582 2

H2B Histone H2B H2B OR HIST2H2BE OR "histone cluster 2 H2B family member e" 3753 2

OR H2BFQ OR H2BGL105 [ti]

H2A Histone H2A H2A OR HIST2H2AC OR "histone cluster 2 H2A family member c" 4306 1

OR "H2A-GL101" [ti]

S100A9 S100 calcium binding S100A9 OR "S100 calcium binding protein A9" 1390 1

protein A9 (calgranulin B) OR MAC387 OR MRP14 OR 60B8AG [ti]

LTF Lactotransferrin LTF OR lactotransferrin OR HLF2 OR GIG12 OR HEL110 [ti] 1052 1

**S1 Table (Cont.).**

**Gene symbol** **Definition Query** **No. of articles No. of articles in**

**in PubMed** **PubMed with NETs**

AZU1 Azurocidin 1 (cationic AZU1 OR "azurocidin 1" OR AZAMP OR CAP37 207 1

antimicrobial protein 37) OR NAZC OR hHBP [ti]

DEFA-1 Defensin, alpha 1 DEFA1 OR "defensin alpha 1" OR DEF1 OR "HNP-1" [ti] 155 1

TKT Transketolase TKT OR transketolase OR HEL107 OR TK1 [ti] 2004 0

S100A8 S100 calcium binding S100A8 OR "S100 calcium binding protein A8" 1381 0

protein A8 OR MRP8 OR MA387 OR 60B8AG [ti]

KRT-10 Keratin 10 KRT10 OR "keratin 10" OR EHK OR K10 [ti] 842 0

ACTB Actin, beta ACTB OR "actin beta" OR BRWS1 OR PS1TP5BP1 [ti] 807 0

ENO1 Enolase 1 ENO1 OR "enolase 1" OR NNE OR MPB1 OR "HEL-S-17" [ti] 796 0

MYH9 Myosin, heavy chain 9, MYH9 OR "myosin heavy chain 9" OR EPSTS [ti] 554 0

non-muscle

ACTN4 Actinin, alpha 4 ACTN4 OR "actinin alpha 4" OR "ACTININ-4" [ti] 286 0

LCP1 Lymphocyte cytosolic LCP1 OR "lymphocyte cytosolic protein 1" OR LC64P 155 0

protein 1 (L-plastin) OR "HEL-S-37" OR "L-PLASTIN" [ti]

MNDA Myeloid cell nuclear MNDA OR "myeloid cell nuclear differentiation antigen" 104 0

differentiation antigen OR PYHIN3 [ti]
